# Exploratory analysis using machine learning to predict for chest wall pain in patients with stage I non‐small‐cell lung cancer treated with stereotactic body radiation therapy

**DOI:** 10.1002/acm2.12415

**Published:** 2018-07-10

**Authors:** Hann‐Hsiang Chao, Gilmer Valdes, Jose M. Luna, Marina Heskel, Abigail T. Berman, Timothy D. Solberg, Charles B. Simone

**Affiliations:** ^1^ Department of Radiation Oncology University of Pennsylvania Philadelphia PA USA; ^2^ Department of Radiation Oncology University of California – San Francisco San Francisco CA USA; ^3^ Department of Radiation Oncology University of Maryland School of Medicine Baltimore MD USA

**Keywords:** chest wall pain, dosimetry, machine learning, non‐small‐cell lung cancer, SBRT

## Abstract

**Background and purpose:**

Chest wall toxicity is observed after stereotactic body radiation therapy (SBRT) for peripherally located lung tumors. We utilize machine learning algorithms to identify toxicity predictors to develop dose–volume constraints.

**Materials and methods:**

Twenty‐five patient, tumor, and dosimetric features were recorded for 197 consecutive patients with Stage I NSCLC treated with SBRT, 11 of whom (5.6%) developed CTCAEv4 grade ≥2 chest wall pain. Decision tree modeling was used to determine chest wall syndrome (CWS) thresholds for individual features. Significant features were determined using independent multivariate methods. These methods incorporate out‐of‐bag estimation using Random forests (RF) and bootstrapping (100 iterations) using decision trees.

**Results:**

Univariate analysis identified rib dose to 1 cc < 4000 cGy (*P* = 0.01), chest wall dose to 30 cc < 1900 cGy (*P* = 0.035), rib Dmax < 5100 cGy (*P* = 0.05) and lung dose to 1000 cc < 70 cGy (*P* = 0.039) to be statistically significant thresholds for avoiding CWS. Subsequent multivariate analysis confirmed the importance of rib dose to 1 cc, chest wall dose to 30 cc, and rib Dmax. Using learning‐curve experiments, the dataset proved to be self‐consistent and provides a realistic model for CWS analysis.

**Conclusions:**

Using machine learning algorithms in this first of its kind study, we identify robust features and cutoffs predictive for the rare clinical event of CWS. Additional data in planned subsequent multicenter studies will help increase the accuracy of multivariate analysis.

## 1 INTRODUCTION

Stereotactic body radiation therapy (SBRT), or stereotactic ablative radiotherapy (SABR), is an increasingly used radiation modality for the treatment of primary early‐stage^1^ and metastatic lung tumors.^2^ SBRT has been shown to provide effective local control with acceptable toxicity.^3^ It is the preferred treatment modality for medically inoperable stage I non‐small‐cell lung cancer (NSCLC) patients, and there is emerging evidence and investigation regarding its role for selected operable NSCLC patients,^4^, ^5^, ^6^ as well as for stage I small‐cell lung cancer patients.^7^, ^8^

The chest wall has been identified as an organ at risk for SBRT, with chest wall toxicities of any grade ranging from 2% to 45% following SBRT.^9^, ^10^, ^11^, ^12^ Radiation‐related chest wall toxicity can result from radiation‐induced rib fracture or chest wall syndrome (CWS). In the absence of rib fracture, CWS is caused by radiation‐induced neuropathy of the intercostal nerves or nerve branches, chest wall edema, chest wall fibrosis, or hairline rib fractures not clearly visible on imaging.^12^, ^13^, ^14^

There is currently a paucity of data on standard dose–volume constraints for the chest wall, with no clear consensus on how to balance target coverage versus chest wall/rib sparing or how factors like fractionation impact CWS. A commonly used constraint is chest wall dose to 30 cc < 30 Gy,^15^ yet there is no prospectively validated data to support this threshold. There have been efforts in recent years to identify the risk factors for rib fractures and CWS and to refine the clinical and dosimetric predictors of chest wall toxicity using dose–response models.^15^, ^16^, ^17^, ^18^

One challenge in evaluating predictive factors for CWS is the low and varying range of events observed.^14^, ^17^, ^19^ Machine learning has previously been used in radiation oncology for a variety of problems, from quality assurance to outcome prediction.^20^, ^21^, ^22^, ^23^, ^24^, ^25^, ^26^ In circumstances where the event being analyzed is relatively uncommon, machine learning algorithms are advantageous in magnifying events. This is achieved by developing models that can learn from and make predictions of a given dataset. Examples include hierarchical clustering models which can iterate quickly through different features and cutoffs in order to identify potentially predictive factors based on how effectively events are separated from nonevents.^26^ The use of these computational algorithms to mine raw data can filter out noise and identify the pertinent factors when the number of events is smaller than the number of features. This current study, the first of its kind, utilizes such algorithms to identify specific dosimetric thresholds predictive for CWS in 197 consecutive patients with Stage 1 NSCLC treated with SBRT.

## 2 MATERIALS AND METHODS

### 2.A Patient inclusion

This study was approved by our institutional review board. A cohort of 197 consecutive patients diagnosed with Stage I NSCLC and treated with SBRT from June 24, 2009, to July 31, 2013, to allow for adequate toxicity follow‐up was identified. All patients were treated to a biologically effective dose (BED) of ≥ 100 Gy in one of four fractionation schemes: (a) 20 Gy × 3 fractions, 12.5 Gy × 4 fractions, 10 Gy × 5 fractions, or 7.5 Gy × 8 fractions. All patients were planned with a constraint goal to keep 30 cc of the chest wall to <30.0 Gy. Twenty‐five parameters (termed features in the machine learning analysis) suspected of a correlation or previously reported^10^, ^12^, ^13^, ^15^, ^17^, ^27^, ^28^, ^29^, ^30^, ^31^ to associate with CWS were analyzed, including patient and tumor characteristics and dosimetric features were recorded for each patient. Toxicities were assessed using CTCAEv4 criteria for chest wall pain, where Grade 1 represents mild pain, Grade 2 represents moderate pain limiting instrumental activities of daily living (ADL), and Grade 3 represents severe pain limiting self‐care ADL.

### 2.B Feature definition

Twenty‐five features were analyzed in this study. They were classified in two subsets: (a) highly important features (*n* = 10) and (b) important features (*n* = 15) by a thoracic radiation oncologist specializing in lung SBRT. Highly important features were features judged to likely correlate to CWS based on the published literature and clinical judgment. The important features group included those hypothesized to be potentially clinically correlated with CWS without any appreciable prior published data. The evaluated features (Table 1) were as follows.

**Table 1:** List of all features selected for analysis. Each feature selected for analysis is listed and broken down by classification as a highly important or important feature

Highly important features	Chest wall dose to 30 cc
	Rib dose to 1 cc
	Rib dose max
	Medically inoperable vs patient refusal
	Dose per fraction
	Age
	Body mass index
	Tumor size (cm)
	PTV volume (cc)
	Age at first fraction
Important features	Total dose
	Diabetes (Y/N)
	Diffusion capacity of the lung for carbon monoxide (DLCO adj%)
	Forced expiratory volume (FEV1(L))
	Decadron/prednisone use
	TNM status
	Stage
	Histology
	Lung mean dose
	Lung dose to 1000 cc
	Lung volume receiving 10 Gy
	Number of fractions

#### 2.B.1 Highly important features

Highly important features consist of chest wall dose to 30 cc, rib dose to 1 cc, rib dose max, medically inoperable versus patient refusal, dose per fraction, age, body mass index, tumor size (cm), PTV volume (cc), and age at first fraction.

#### 2.B.2 Important features

Important features consist of total dose, diabetes (Y/N), diffusion capacity of the lung for carbon monoxide (DLCO adj%), forced expiratory volume (FEV1(L)), decadron/prednisone use, TNM status, stage, histology, lung mean dose, lung dose to 1000 cc, lung dose to 1500 cc, lung volume receiving 20 gy, lung volume receiving 15 gy, lung volume receiving 10 gy, and number of fractions.

All dosimetric indices were calculated with heterogeneity corrections, using the analytical anisotropic algorithm (AAA), Eclipse Version 11.0 (Varian Medical Systems, Palo Alto, CA, USA).

### 2.C Univariate analysis

Univariate CWS thresholds for each feature collected were generated to split the patient population into high‐ and low‐risk subpopulations. These thresholds were determined using decision stumps (simple univariate thresholds) implemented in Matlab R2015a (MathWorks Inc., Natick, MA, USA). In all cases, the deviance was used to measure how far the decision tree is from the target output. It is a smoother version of the classification error and provides a measurement of the quality of the description provided.^32^ Each threshold was characterized by the probability of splitting out patients with and without CWS into the appropriate subpopulations. In addition, a generalization score was determined for each threshold, which was defined as the ratio of true positives for out‐of‐sample to in‐sample data. A cutoff of >0.75 was used for the generalization score, meaning a similar split of the data would result at least 75% of the time. The generalization score is used to characterize out‐of‐sample performance of the univariate dosimetric thresholds, and it quantifies how well these thresholds should perform for data that the algorithm has not encountered.^26^ This analysis was performed under the conditional assumption that the true distribution of patients satisfying the threshold is represented by the patients not developing CWS.

### 2.D Multivariate analysis

Two different algorithms were considered: decision trees, for interpretability, and Random forests, for accuracy.^33^, ^34^, ^35^ Decision trees partition the data into a disjoint number of subpopulations and make a constant prediction at each subpopulation. Random forests predict outcomes by averaging the output of hundreds of decision trees.^36^ For specifics about these algorithms, the readers are referred to “The Elements of Statistical Learning,” a comprehensive book about machine learning.^36^ In this work, the complexity of the models for all algorithms was controlled by choosing hyperparameters (global constants that control the complexity of the algorithms such as the number of times data are allowed to be partitioned in a decision tree) that minimized the leave‐one‐out cross‐validation of the deviance. Leave‐one‐out cross‐validation refers to a method where one observation is left out of the dataset, and then performing training on the remaining observations and predicting the observation that the algorithm has not seen. Specifically, the complexity of the decision tree was optimized through the use of minimum number of observations per node (Min Number per Node). Smaller node sizes result in complex trees that do well in explaining the training dataset with which the algorithm is initially presented but may result in suboptimal results with the testing dataset. This hyperparameter controls the number of observations a terminal node must have before attempting a split. As our goal was to identify the thresholds that best predict CWS in future patients, we tested various training sets (10 training sets in a 10 K‐fold experiment) in order to select hyperparameter values that minimized the testing error.

Two additional analyses were performed to control for overfitting. First, the Min Number per Node was changed from 50 to 80 in steps of 5. Second, for each hyperparameter, a random subsampling of the patient population was performed where a predefined number of patients ranging from 158 to 197 patients would be randomly selected from the data set. One hundred iterations were performed, and an aggregate decision tree was developed. All features that were selected at least 10% of the time were compared to the maximally selected feature.

The complexity of Random forests was controlled by selecting the maximum number of splits allowed per individual tree and the number of variables randomly subsampled. Unless otherwise specified, 500 individual decision trees were combined when Random forest was used. The default hyperparameter, the square root of the number of features, was used for the number of feature subsamples in Random forests. In all cases, artificial equal prior probabilities, which is where the initial weights of the observations are up‐sampled for the minority event (e.g., development of CWS) and under‐sampled for the majority event (e.g., absence of CWS), such that their sum would be equal, were used to avoid the inherent bias in the algorithms due to the skewed dataset. One hundred iterations were performed, and we identified features that have an out‐of‐bag importance, which is at least 10% of the maximally selected feature. The out‐of‐bag importance, as defined by Breiman, is an unbiased estimator of the predictive value of a feature, which uses randomly generated training sets by sampling with replacement.^33^, ^34^

### 2.E Learning‐curve experiments

In order to test the self‐consistency of our data, Learning‐curve experiments were performed. In a Learning‐curve experiment, different numbers of patients are subsampled from the original dataset, models are built using the subsampled data assumed to be training data, and then the training and testing errors are determined.

## 3 RESULTS

### 3.A Patient characteristics and identification of features and thresholds predicting CWS

Twenty patients were treated with a 20 Gy × 3 fractions regimen, 102 patients were treated using a 12.5 Gy × 4 fractions, 66 patients were treated using 10 Gy × 5 fractions, and 12 patients were treated using a 7.5 Gy × 8 fractions regimen. In our dataset, 11 patients developed Grade ≥2 chest wall toxicity. A univariate analysis to identify optimal patient separation thresholds for CWS development was performed on the initial set of 10 highly important and 15 important patient features. This analysis revealed three Highly important features, including chest wall dose to 30 cc (*P* = 0.035), rib dose to 1 cc (*P* = 0.01), and rib dose max (*P* = 0.05), and one important feature [lung dose to 1000 cc (*P* = 0.039)], as statistically significant (Table 2). These four features and their corresponding thresholds all met a generalization score of >0.75.

**Table 2:** Significant features identified on univariate analysis. Features with a CWS threshold with *P* < 0.05 (without adjustment for multiple comparisons) and generalization value > 0.75. The number of patients in each subgroup by feature threshold and the number and percentage of patients developing CWS in each subgroup are listed for reference. All features had missing values; therefore, the number of patients is <197 for each

Feature	Thresholds	Subpopulations risks^a^	*P* value
Rib dose to 1 cc	<4000 cGy	(*N* = 83, 1 CWS event; 1.2%) vs (*N* = 80, 8 CWS events; 10.0%)	0.010
Chest wall dose 30 cc	<1900 cGy	(*N* = 44, 0 CWS events; 0%) vs (*N* = 134, 11 CWS events; 32.4%)	0.035
Lung dose to 1000 cc	<70 cGy	(*N* = 70, 1 CWS event; 1.4%) vs (*N* = 126, 10 CWS events; 7.9%)	0.039
Rib Dmax	<5100 cGy	(*N* = 65, 1 CWS event; 1.5%) vs (*N* = 98, 8 CWS events; 8.2%)	0.050

a Patient numbers do not add to 197 due to missing values present for select parameters.

### 3.B Decision tree modeling

**Figure 1:**
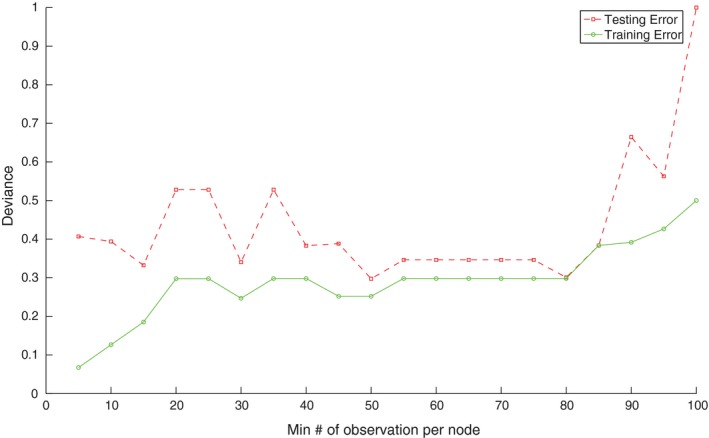
Modeling of optimal node size. The testing and training error for the dataset based on different hyperparameter settings of Min Number of observations per node using a leave‐one‐out cross‐validation of the deviance.

The decision tree analysis revealed that when evaluating different node sizes ranging up to 100 patients per terminal node, values of this hyperparameter ranging from 50 to 80 patients per terminal node produce similar results in the testing dataset, with local minima observed at 50 patients per node and 80 patients per node (Fig. 1). When evaluating terminal node sizes above 80 patients per node, the decision trees become overly simplified, resulting in the training and testing dataset errors being similar.

**Figure 2:**
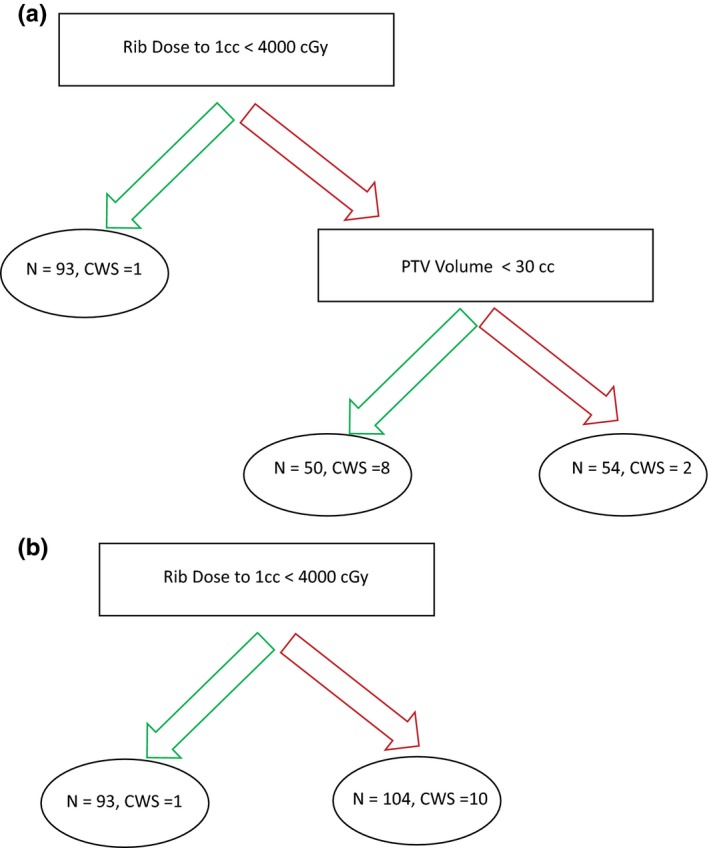
Decision tree examples based on differential minimum node size. (a) Decision tree using a Min Number per Node = 50. (b) Decision tree using a Min Number per Node = 80.

Decision trees with node sizes of 50 and 80 patients per terminal node are shown in Fig. 2. In the scenario where the Min Number per Node = 50, our machine learning algorithm identifies rib dose to 1 cc < 4000 cGy as an important feature and dose threshold, with only one of 93 (1.1%) patients below this threshold developing CWS, as compared with 10 of 104 (9.6%) patients exceeding this threshold. With these parameters, a second split was generated, demonstrating that a smaller PTV volume is associated with a higher incidence of CWS [Fig. 2(a)]. If instead, the Min Number per Node = 80, only the first split is obtained [Fig. 2(b)].

### 3.C Feature robustness and data consistency

**Figure 3:**
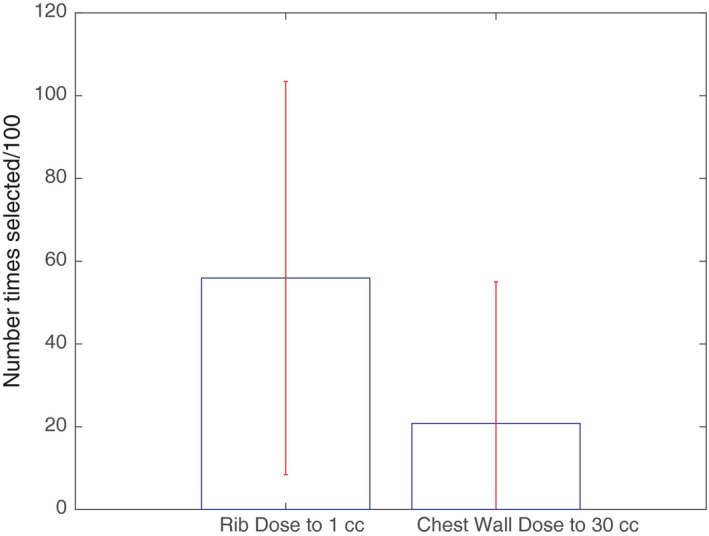
Multivariate analysis using hyperparameter adjustment and multiple iterations. Number of times features selected for 100 iterations and different hyperparameters. Min Number per Node = 50–80. In each iteration, 10–40 patients (in increments of 2) were randomly left out to introduce variation on the resulting decision tree. Features with a mean value equal to 0.1 of the feature that is selected the most are shown. The top of each bar represents the number of times each feature is selected per 100 iterations. Error bars represent 1 SD.

**Figure 4:**
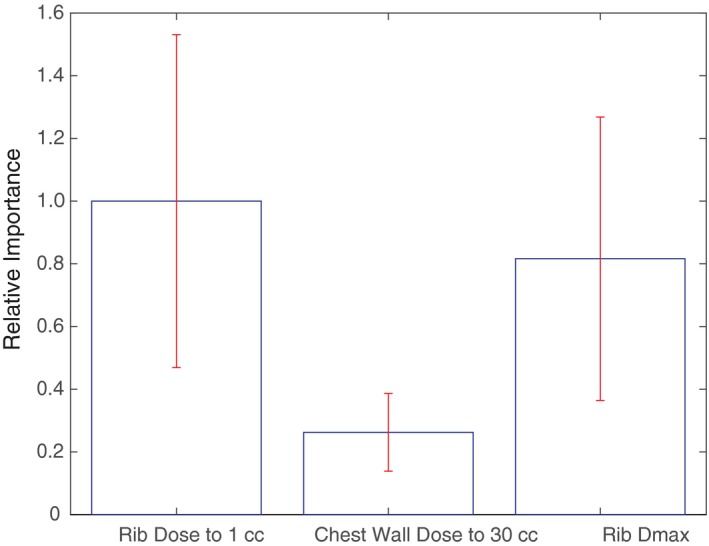
Multivariate analysis using Random forests. Relative out‐of‐sample importance of different features calculating using Random forests. The top of the each bar represents the magnitude of out‐of‐sample importance for each feature. Error bars represent 1 SD.

When introducing variation components of differing nodal size and random subsampling of the population to test for feature robustness, only rib dose to 1 cc and chest wall dose to 30 cc were selected as features that influence development of CWS (Fig. 3). Random forest analysis performed as part of a second and separate analysis of robustness also identified rib dose to 1 cc and chest wall dose to 30 cc as predictors of CWS. Rib Dmax was additionally identified as a potential predictor for CWS (Fig. 4), whereas PTV volume was excluded.

**Figure 5:**
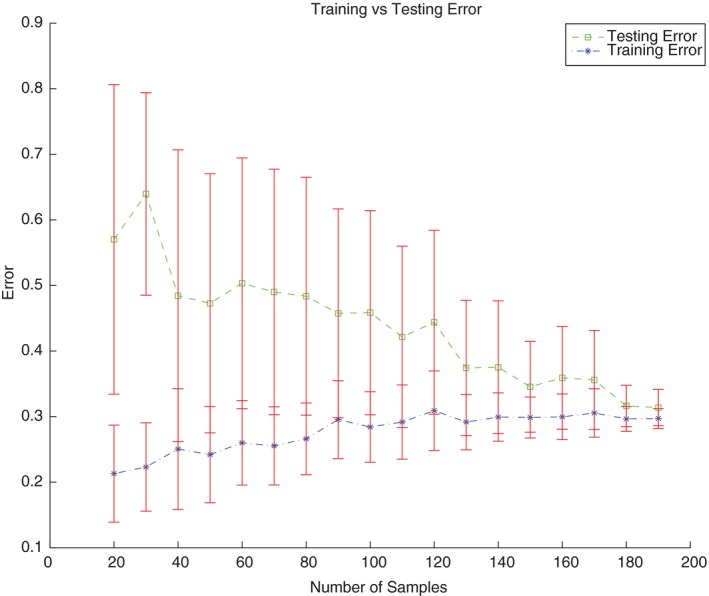
Learning curve for analysis dataset. Out‐of‐sample deviance estimated for training and testing data for different number of observations used for training. All points refer to the mean value over 100 iterations.

Using learning‐curve experiments with different hyperparameters, we found that as patient number in the training set increases, the training error increased and the testing error decreased. The learning‐curve experiments established that our patient dataset is likely to provide a true representation of the wider population with regard to developing CWS (Fig. 5). This data consistency verification confirms accepting the previously identified CWS predictors of rib dose to 1 cc < 4000 cGy, chest wall dose to 30 cc < 1900 cGy, and rib Dmax < 5100 cGy (all *P* < 0.05).

## 4 DISCUSSION

There is scarcity of clear literature and guidance on dosimetric constraints for CWS. This study used a cohort of 197 consecutive patients with Stage I NSCLC treated with SBRT to identify clinically relevant predictive features. In this cohort, 11 (5.6%) patients developed CTCAE v4 grade ≥2 chest wall pain. Prior studies suggest rates of CWS, or chest wall pain without evidence of rib fracture, range from 2% to 8%^14^, ^17^, ^19^ after SBRT, consistent with our observations. Conventional analyses struggle to identify meaningful clinically relevant thresholds for predicting toxicity due to the low number of absolute events. Machine learning algorithms are generally well suited to this challenge, given their advantages in parsing large datasets in order to robustly stratify out rare events.^37^

Prior efforts investigating predictors for chest wall toxicity are generally conflicting or inconclusive on the relative importance of risk factors, likely due to the limited number of patients and events assessed. A subset of prior studies of chest wall toxicity^15^, ^16^ have suggested that within the commonly used total dose and fractionation schemes for SBRT, dosimetric predictors do not result in significantly different rates of toxicity. However, this does not imply that rib and chest wall doses can be simply disregarded since (a) CWS can result without the clinical appearance of rib fractures and (b) increasing risk may still occur with increasing dose in a continuous fashion.^18^ Other prior studies have examined factors including rib Dmax, rib dose to 0.5 cc, 2 cc, rib V30, V40, chest wall Dmax, chest wall dose to 8 cc, and chest wall V30, without clear indication of the relative superiority or inferiority of one of these dosimetric factors relative to the others.^9^, ^13^, ^27^, ^29^, ^38^, ^39^ Global Dmax and fraction size are also suggested to be important indicators of toxicity, with higher rates of radiation‐induced rib fracture of ~50% for DMax > 54 Gy and fraction size >8 Gy.^16^ Regarding the chest wall specifically, constraints of V30 Gy < 70 cc,^12^ V30 Gy < 35 cc,^13^ and D30 cc < 30 Gy have been recommended.^15^ Kimsey et al. examined chest wall tolerance in SBRT and suggest that the D70 cc is an important factor to consider dosimetrically.^15^

In this study, we analyzed 25 patient features, both dosimetric and nondosimetric. Similar to the published literature, we found that rib dose^29^ and chest wall dose^13^ are important dosimetric features. Decision trees were considered the baseline algorithm because of the ability to produce models that are clinically interpretable and could be validated according to prior clinical knowledge. Random forest was used to evaluate feature importance and to generate and explore additional hypotheses. By separating the data into training (data used to create the models) and testing (data used to evaluate the performance of the model and not seen during training) proper estimation of the error expected for the algorithm could be established. Training and testing errors refer to errors calculated on these datasets. In addition, by using interpretable algorithms like decision trees (those that produced models that clinical practitioners can understand) and black box algorithms (those that produce models that cannot be easy understood but are potentially more accurate) like Random forests that combine the input of hundreds of trees into one prediction different hypothesis and important features can be automatically selected.^33^, ^35^, ^36^, ^40^ If the data are self‐consistent, then the training error increases along with the number of patients used to build the model. Conversely, if true knowledge is acquired from the data, then the testing error will decrease with the number of patients used for training.

Our final model, which combines the results of the baseline analysis using decision trees and is supplemented by the results of Random forests, specifically identify a cutoff of rib dose to 1 cc < 4000 cGy, chest wall dose to 30 cc < 1900 cGy, and rib Dmax < 5100 cGy as important prognosticators (Table 2, Figs. 3 and 4). As the large majority of the patients in the data set were treated using 10 Gy × 5 or 12.5 Gy × 4 fractionation schemes (85%), the dosimetric predictors derived from this study are most applicable to patients treated with either of these regimens.

While machine learning is a potentially powerful tool, indiscriminate use also has the potential to result in erroneous or invalid results. In this study, we employ different hyperparameter settings and utilize several different algorithms to validate findings and filter out spurious results. Through use of multiple permutations, random subsampling, variation in node size, and robust decision tree modeling, we identified the features that are likely to truly contribute to the development of CWS. These data verification methodology allowed us to filter out an initial result, suggesting lower PTV volumes contributed to increased CWS development [Fig. 2(a)]. Our analysis also reliably reproduced rib dose to 1 cc as an important feature, as well as confirming chest wall dose to 30 cc and rib Dmax as robust features (Figs. 3 and 4).

Our findings are consistent with prior analyses illustrating the importance of chest wall dose, but this analysis utilizes the added advantage of machine learning to assign increased importance to a specific chest wall dosimetric factor. This has the potential to allow for informed, evidence‐backed clinical decision making in scenarios where two or more planning goals may be mutually exclusive and priority must be given to one. A prior study by Thibault et al.^17^ suggests that location alone predicts for rib fracture, with the incidence increased in peripherally located tumors. Their efforts showed no significant predictive dosimetric criteria, with the only other predictive clinical factor being the presence of osteoporosis. Our data confirm the importance of tumor location, as it follows that radiation dose in proximity to the chest wall and ribs are necessary for the development of CWS. However, our findings suggest that tumor location may be a surrogate for dose received by the ribs and chest wall, which are the true drivers of the development of CWS. Due to the relative rarity of events, our machine learning approach may have allowed for us to identify potentially relevant dosimetric factors that were not identified by Thibault et al.

A potential shortcoming of this study, and other similar studies, lies with the fact that CWS grading is inherently subjective. This is a potential bias intrinsic to analyses of CWS. Our study is likewise unable to compensate for this underlying subjectivity. Another limitation of our study is that an exhaustive analysis of all possible variables and thresholds is prohibitive, despite utilizing machine learning. With the chest wall constraints, we evaluated the commonly employed constraint of chest wall dose to 30 cc. A weakness of this approach is whether this choice represents the ideal volumetric constraint. Future investigations assessing continuous volumetric modeling of the chest wall constraint in addition to continuous dose modeling are warranted. This could likewise be employed to other relevant thoracic structures like the rib, akin to a prior effort by Petterson et al.,^29^ and lung dose. Expanded datasets in future analyses will add to the robustness of our findings, and future work will focus on external validation in a multicenter analysis.

## 5 CONCLUSIONS

The strength of this study, the first of its type, is in the use of machine learning heuristic clustering analysis to identify factors in a continuous fashion that would predict both for and against CWS by incorporating patient‐ and tumor‐related variables and dosimetric factors. From our analysis, we conclude that in patients treated with SBRT using common and standard fractionation schemes (4 × 12.5 Gy, 5 Gy × 10), providers should attempt to keep the rib dose to 1 cc <4000 cGy, chest wall dose to 30 cc < 1900 cGy, and rib Dmax < 5100 cGy in order to mitigate CWS. These novel and clinically meaningful metrics provide a guide for treatment planning of SBRT and contribute to the knowledge base for patient counseling and informed consent.

## CONFLICT OF INTEREST

We do not have any conflicts or potential conflicts of interest to disclose at this time. We do not use any copyrighted information or patient photos. The data presented in this manuscript was acquired in accordance with the policies of the institutional review board at our institution.
